# Risk Prediction of Coal and Gas Outburst in Deep Coal Mines Based on the SAPSO-ELM Algorithm

**DOI:** 10.3390/ijerph191912382

**Published:** 2022-09-28

**Authors:** Li Yang, Xin Fang, Xue Wang, Shanshan Li, Junqi Zhu

**Affiliations:** School of Economic and Management, Anhui University of Science and Technology, Huainan 232001, China

**Keywords:** deep coal mine, coal and gas outburst, risk prediction, SAPSO, extreme learning machine algorithm

## Abstract

Effective risk prevention and management in deep coal mines can reduce the occurrences of outburst accidents and casualties. To address the low accuracy and inefficiency of coal–gas outburst prediction in deep coal mines, this study proposes a deep coal–gas outburst risk prediction method based on kernal principal component analysis (KPCA) and an improved extreme learning machine (SAPSO-ELM) algorithm. Firstly, high-dimensional nonlinear raw data were processed by KPCA. Secondly, the extracted sequence of outburst-causing indicator principal components were used as the input variables for the simulated annealing particle swarm algorithm (SAPSO), which was proposed to optimize the input layer weights and implied layer thresholds of the ELM. Finally, a coal and gas outburst risk prediction model for a deep coal mine based on the SAPSO-ELM algorithm was developed. The research results show that, compared with the ELM and PSO-ELM algorithms, the SAPSO-ELM optimization algorithm significantly improved the accuracy of risk prediction for coal–gas outbursts in deep coal mines, and the accuracy rate was as high as 100%. This study enriches the theory and methods of safety management in deep coal mines, and effectively helps coal mine enterprises in improving their ability to manage coal–gas outburst risks.

## 1. Introduction

Coal and gas outbursts are essentially caused by a process of stress concentration, strength destruction, sudden destabilization, and rapid ejection under the influence of gas coupled with a coal body and gas with adsorption properties [1,2]; it is a significantly complex natural disaster [3]. As the world’s top coal-producing and consuming country, China was at 56% of its primary energy consumption in 2021. Its natural resources are “rich in coal, poor in oil, and low in gas”, making it one of the most concerned countries in the world regarding prevention of coal–gas outburst [4,5,6]. The coal-mining depth is increasing at a rate of 10–25 m/a, owing to the gradual depletion of shallow coal resources. Based on statistics, the current deep (below 1000 m) coal resources constitute approximately 53% of the overall coal resources; there are 47 mines deeper than 1000 m that have a maximum mining depth of 1500 m, and more than 30 new 1000 m-deep mines will be built in the next 5–10 years [7]. Additionally, the geological conditions of deep coal seams in China are extremely complex, which threatens the safe coal mine production and safety of miners [8,9]. Therefore, the accurate prediction of coal–gas outbursts in deep coal mines is of great significance for ensuring the safety of deep-seated mine operations [10].

Many scholars globally have launched numerous studies on the issue of coal–gas outburst risk predictions. Acoustic emission and microseismic-monitoring methods are considered the two most common dynamic continuous methods that can effectively predict coal–gas outbursts [11,12,13]. Liu et al. [14] combined a gradient boosting decision tree with the KNN algorithm and proposed an improved classifier model with high prediction accuracy. Support vector machines have evident advantages in predicting small-sample gas outbursts, but they can easily fall into local optima; however, scholars have used the adaptive particle swarm algorithm and Boruta method to optimize it and achieved good prediction results [15,16,17]. Ru et al. [18] first proposed the use of correlation coefficients to fill missing data and exception problems, which effectively solved the data problem and provided a new path for similar coal–gas outburst prediction problems. Digital twins can effectively solve such complex problems, and Wang [19] predicted the coal–gas outburst intensities by conducting experiments based on digital twins with deep learning. Li et al. [20] combined traditional methods with artificial intelligence to propose a multisource information fusion model that provided technical support for safe coal mine production. Furthermore, Fisher’s prediction methods [21], Bayesian discriminant analysis [22], and simulated annealing genetic algorithms [23,24] have been used. Owing to the incredible complexity of deep coal mine gas outbursts, coupled with the increasing mining depth and intensity, there are highly complicated nonlinearities and correlations among the index systems, most of which are based on some of these elements and significantly affect the accuracy of coal–gas outburst prediction in deep coal mines [25,26]. With the increasing maturity of computer technology, deep learning, machine learning, and intelligent algorithms began to be gradually applied to disaster evaluation and prediction. The more commonly used intelligent algorithms in coal and gas outburst evaluation are artificial neural networks (ANN), support vector machines (SVM), and extreme learning machines (ELM). ANN can use the optimal training principle to compute repeatedly and continuously debug the neural network structure until it obtains relatively stable results, achieving dynamic tracking comparison and deep learning. However, the most severe problem is that it cannot explain its inference process and inference basis. SVM is a new method for small-sample learning with a solid theoretical foundation and excellent generalization capability capacity. However, it is difficult to solve multi-modal classification problems, challenging to achieve large-scale training samples, and easy to fall into the local optimum trap. Compared with them, the ELM algorithm randomly generates the connection weights between the input and hidden layers and the thresholds of hidden neurons. There is no need to adjust them during the training process to obtain the unique optimal solution by setting the number of hidden layers. The ELM algorithm has a faster learning speed and good generalization performance. Therefore, to achieve efficient and safe mining in deep mines, it is necessary to understand the system of outburst-inducing factors and conduct in-depth research to prevent and handle gas outburst disasters more effectively.

The ELM is a new single hidden layer feedforward neural network that can sufficiently handle multimodal classification problems such as deep coal mine gas outburst prediction. Its input layer weights and implied critical layer values are usually set unexpectedly and are not sufficiently stable. The PSO focuses on cooperation among particles by simply adjusting the behavior patterns of individual particles to achieve optimal solutions to complex problems. However, it also has the disadvantages of not sufficiently converging quickly and is prone to premature convergence. The SA can enhance the particle swarm diversity, effectively suppress the occurrence of premature convergence, and improve the algorithm’s global search capability. This study therefore proposes a coal–gas outburst prediction model for deep coal mines based on kernal principal component analysis (KPCA) and an improved extreme learning machine (SAPSO-ELM) algorithm. The KPCA can handle high-dimensional nonlinear raw data. The extracted sequence of outburst indicators was used as the input variable for the improved ELM model. The simulated annealing particle swarm algorithm (SAPSO) was proposed to optimize the input layer weights of the ELM and implied layer thresholds, which dramatically improved the prediction accuracy and reliability of the ELM model. Through example validation and comparative analysis, it is proven that the model has a strong generalization ability.

## 2. Characteristics Extraction of Factors Affecting Coal and Gas Outburst in Deep Coal Mines

### 2.1. Selection of Mutagenic Factors

The mechanism of coal and gas outbursts in deep coal mines is complex [27,28], and there are multiple hazard couplings [29]. However, integrated action theory has become an academic consensus. This theory suggests that coal–gas outbursts are caused by the combination of gas pressure, ground stress, and physical properties of the coal rock body [30,31]. To further improve the accuracy of coal–gas outburst prediction in deep coal mines, we constructed a new system in this study, which is based on an in-depth analysis of coal–gas outburst mechanisms and influencing factors, combined with examples of coal–gas outburst accidents in deep coal mines, and the existing literature on outburst prediction indices [21,32,33,34,35]. The new system, which is also an improvement on the original gas outburst prediction system, contains coal thickness variability coefficient X_1_, coal damage type X_2_, lithology X_3_, mining depth X_4_, mine temperature X_5_, soft stratification thickness X_6_, original gas pressure X_7_, original gas content X_8_, resolvable gas content X_9_, coal seam permeability coefficient X_10_, gas discharge initial velocity X_11_, coal solidity coefficient X_12_, and 12 other deep mine gas outburst prediction indices. Many factors affect coal–gas outbursts in deep coal mines, and complex coupling and nonlinearity exist among the outburst-causing factors. To select as many representative indicators as possible, the initial data should be retained to the greatest extent, and the correlation between indicators should not be large. This study selected KPCA to integrate the information of predicted indicator data, extract the principal components of indicators, simplify the model structure, and improve prediction accuracy.

A total of 178 sets of raw data were obtained, as detailed in Table 1, which were divided into five categories (1–5) based on the coal damage type X_2_—undamaged, generally damaged, lightly damaged, strongly damaged, and damaged to powder; they were also classified based on four types of lithology X_3_—strong, stronger, average, and softer—represented by numbers 1–4. The higher the grade, the more likely it is to be prominent. The rest were objectively measured or objective data were derived from the measured data. Except for the coal seam permeability coefficient X_10_ and coal firmness coefficient X_12_, which are negative indicators, all others are positive. In this study, 1, 2, 3, and 4 were used to indicate the degree of possible coal–gas outburst risk levels in the mine, where 1, 2, 3, and 4 indicate that no coal–gas outburst will occur (0 m^3^/t), the risk of coal–gas outburst disasters is small (0–5 m^3^/t), coal–gas outburst disasters will generally occur (5–10 m^3^/t), and serious coal–gas outburst disasters will occur (10 m^3^/t or more), respectively.

### 2.2. Kernel Principal Component Analysis (KPCA)

KPCA [36,37] is mainly used to address nonlinear complex problems. It essentially employs a nonlinear function with extremely high nonlinear-processing capability, through which the introduced function maps a large amount of complex information in the original space to linearly divisible high-dimensional data. This function is widely used in fields such as dimensionality reduction in indicator features to extract the main information features, reduce information redundancy, and minimize the number of operations. In practical problem processing, Gaussian radial basis kernel functions are favored by scholars owing to their superior processing performance and outstanding anti-interference ability [38].

$X={\left[x_{1},x_{2},\cdots ,x_{n}\right]}^{T}\in R^{n*m}$ is the initial dataset, where *n* represents the total number of samples, and m represents the data dimensionality. The corresponding operation can be completed for the data points $x_{1},x_{2},\cdots ,x_{n}$ to transform them into a certain feature space *F* using the nonlinear function Φ. At this point, $\Phi \left(x_{k}\right)$ can be used for representation, and the covariance matrix formed within *F* is shown in Equation (1):

(1) $$C^{F}=\frac{1}{N}\sum_{i=1}^{N}\Phi \left(x_{i}\right)\Phi {\left(x_{i}\right)}^{T}$$

If the eigenvalues and eigenvectors of *C^F^* are expressed in terms of *λ* and *v*, respectively, their relationship can be expressed by Equation (2):

(2) $$C^{F}v=\lambda v$$

In Equation (2), $\lambda =\left[\lambda_{1},\lambda_{2},\ldots ,\lambda_{N}\right]$ is an eigenvalue matrix obtained by solving for *C^F^* with each eigenvalue satisfying *λ*_1_ ≥ *λ*_2_ ≥, …, ≥ *λ_N_*, and *α_i_* (*i* = 1, 2, … *M*) represents the existence coefficient of a term, and this coefficient can provide some basis for the establishment of Equation (3):

(3) $$v=\sum_{i=1}^{N}\alpha_{i}\Phi \left(x_{i}\right)$$

Substituting this into Equation (1) and multiplying Φ(*x_k_*) on both sides of Equation (1) yields:

(4) $$\lambda \left(\Phi \left(x_{k}\right)\sum_{i=1}^{N}\alpha_{i}\Phi \left(x_{i}\right)\right)=\Phi \left(x_{k}\right)C^{F}\left(\sum_{i=1}^{N}\alpha_{i}\Phi \left(x_{i}\right)\right)$$

A simplified operation of Equation (4) yields:

(5) Nλα=Kα

In Equation (5), *K* represents the *N* × *N* dimensional kernel matrix, where *K_jk_* = *K*(*x_j_*,*x_k_*) = Φ(*x_j_*)*^T^*Φ(*x_k_*), and *α* = [*α*_1_, *α*_2_, …, *α*_n_]*^T^* represents the associated eigenvectors in the kernel matrix K.

Before performing KPCA, matrix *K* is centralized by replacing $\bar{K}$ in Equation (5):

(6) $$\bar{K_{\mathrm{ij}}}=\left(\Phi \left(x_{i}\right)-\frac{1}{N}\sum_{m=1}^{N}\Phi \left(x_{m}\right)\right)\left(\Phi \left(x_{j}\right)-\frac{1}{N}\sum_{n=1}^{N}\Phi \left(x_{n}\right)\right)={\left(K-1_{N}K-K1_{N}+1_{N}K1_{N}\right)}_{ij}$$

In Equation (6), 1*_N_* represents a type of *N* × *N* matrix in which each factor is 1/*N*, and *N* is the dimension of *K*.

Through Equations (5) and (6), it is possible to complete the calculation of the eigenvector v of matrix *C^F^* based on the eigenvector *α* of the *K* matrix. Subsequently, the cumulative variance contribution ratio generated by the relevant eigenvalues is calculated using Equation (7), and the top *P* eigenvalues are used to study based on the contribution ratio requirement. Simultaneously, the determination of the total number of principal components by data feature extraction can be realized based on these eigenvalues.

(7) $$L\left(p\right)=\sum_{k=1}^{p}\lambda_{k}/\sum_{k=1}^{N}\lambda_{N}$$

## 3. SAPSO Optimized ELM-Based Coal and Gas Outburst Prediction Model for Deep Coal Mines

### 3.1. Extreme Learning Machine Prediction Model

The extreme learning machine (ELM) is a new single hidden layer feedforward neural network that can sufficiently handle multimodal classification problems such as deep coal mine gas outburst prediction. It maintains many advantages, such as high learning accuracy of general neural networks that improves the learning efficiency to a great extent, optimizes the gradient descent-based learning, straightforward computational process, strong generalization power, and it has been popularized in several industries and disciplines [39,40,41].

**Figure 1:**
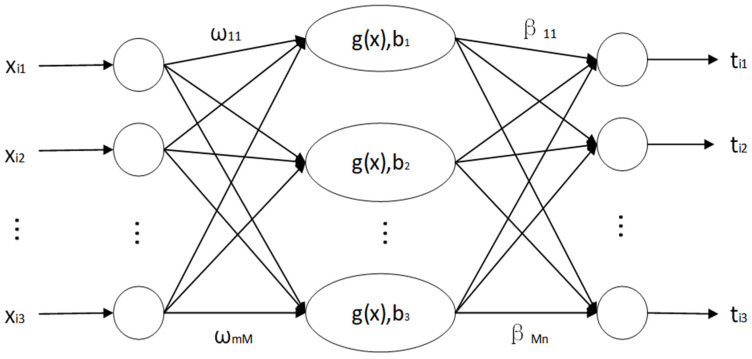
Schematic of extreme learning machine network training model.

The forward single hidden layer architecture in ELM is chosen as the network training model. The number of nodes in the three parts of the network, such as the input layer, hidden layer, and output layer, are represented by *m*, *M*, and *n*, respectively; *g*(*x*) represents the activation function involved in the hidden layer neurons, and bi is the critical value. Assuming that the number of samples (*x_i_*,*t_i_*) with differentiation is *N*, 1 ≤ *I* ≤ *N*, the ELM structural model is as shown in Figure 1.

The ELM structural model is mathematically expressed as follows:

(8) $$\sum_{i=1}^{M}\beta_{i}g\left(\omega_{i}x_{i}+b_{i}\right)=o_{j},j=1,2,\ldots ,N$$

In Equation (8), $\omega_{i}=[\omega_{i1},\omega_{i2},\omega_{i3},\ldots ,\omega_{im}]$ represents the input weight vector that connects both the input layer node and *i*-th hidden layer node, $\beta_{i}={\left[\beta_{i1},\beta_{i2},\beta_{i3},\ldots ,\beta_{in}\right]}^{T}$ represents the output weight vector that connects the output layer to the hidden layer nodes, and $o_{i}={\left[o_{i1},o_{i2},o_{i3},\ldots ,o_{in}\right]}^{T}$ represents the network output value.

For the extreme learning machine, the cost function *E* can be expressed through the following equation:

(9) $$E\left(S,\beta \right)=\sum_{j=1}^{N}\left\|o_{j}-t_{j}\right\|$$

In Equation (9), $S=(w_{i},b_{i},i=1,2,\ldots ,M)$ involves the input weights and hidden node critical values. The learning objective of the ELM computational model is to search for the ideal *S* and *β*, such that the error between the output and actual results is as small as possible, that is, min(*E*(*S*,*β*)).

(10) $$\min\left(E\left(S,\beta \right)\right)=\underset{\omega_{i},b_{i},\beta }{\min}\left\|H\left(\omega_{1},\ldots ,\omega_{M},b_{1},\ldots ,b_{M},x_{1},\ldots ,x_{N}\right)\beta -T\right\|$$

In Equation (10), *H*, *β*, and *T* represent the output matrix of the hidden layer, *β* output weight matrix, and target value matrix, respectively, and the formula defining *H*, *β*, and *T* can be expressed by the following equations:

(11) $$H\left(\omega_{1},\ldots ,\omega_{M},b_{1},\ldots ,b_{M},x_{1},\ldots ,x_{N}\right)={\left[\begin{array}{l} g\left(\omega_{1}x_{1}+b_{1}\right)\cdots g\left(\omega_{M}x_{1}+b_{M}\right)\\ \vdots \\ g\left(\omega_{1}x_{N}+b_{1}\right)\cdots g\left(\omega_{m}x_{N}+b_{M}\right) \end{array}\right]}_{N\times M}$$

(12) $$\beta ={\left[\begin{array}{l} \beta_{1}^{T}\\ \vdots \\ \beta_{M}^{T} \end{array}\right]}_{M\times N},T={\left[\begin{array}{l} t_{1}^{T}\\ \vdots \\ t_{N}^{T} \end{array}\right]}_{N\times N}$$

The ELM computational model essentially involves the process of solving the ideal solution of a nonlinearly varying target object. During this period, the process can be transformed into a least-squares solution-related computation for the minimum parametrization of the linear system Hβ=T:

(13) $$\hat{\beta }=H^{+}T$$

In Equation (13), *H*^+^ represents the MP generalized inverse of matrix *H*. This is the ELM training process.

### 3.2. Simulated Annealing Particle Swarm Algorithm (SAPSO)

**Figure 2:**
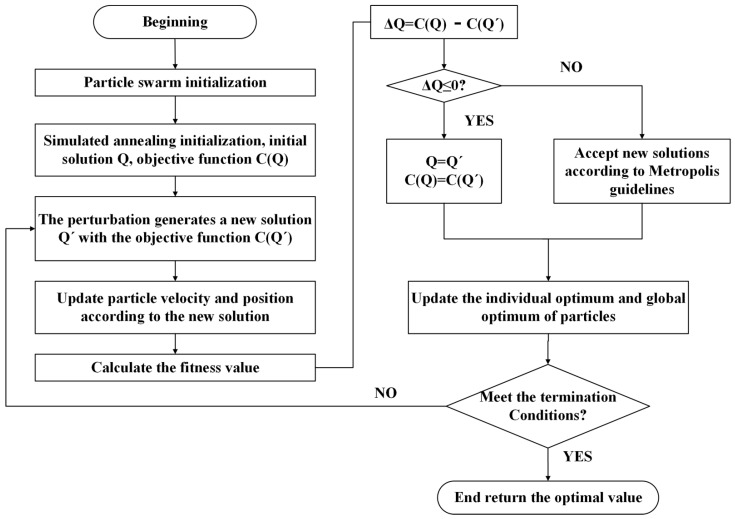
SAPSO algorithm flow chart.

Particle swarm optimization (PSO) is a swarm intelligence optimization algorithm proposed by American scholars James Kennedy and Russell Eberhart in 1995 [42,43], which focuses on the cooperation among particles by simply adjusting the behavior patterns of individual particles to achieve optimal solutions to complex problems. Particle swarm algorithms have the advantages of few adjustment variables, fast operation, and high accuracy, and are used in many fields, including optimization functions and machine learning [44]. However, it also has the disadvantages of not sufficiently converging quickly, and is prone to premature convergence; this study uses simulated annealing (SA) to improve it and obtain the simulated annealing particle swarm algorithm (SAPSO), as shown in Figure 2.

The SA algorithm [45,46], in seeking the optimal solution to the target problem, first determines the initial temperature and interferes with it, simulates the energy state transfer process of the particle at different temperatures, compares the new solution with the current key, and replaces it based on the Metropolis criterion. The SA algorithm accepts the optimal solution with probability 1 and agrees with the worst solution with some possibility as a way to escape the local optimal solution trap. The Metropolis criterion defines the probability of internal energy formation when an object is in a specific temperature T environment, and gradually transforms from states *i* to *j*, which is expressed by the following equation:

(14) $$p_{ij}^{T}=\{\begin{array}{cc} 1 & \left(E\left(j\right)\leq E\left(i\right)\right)\\ e^{-\frac{E\left(j\right)-E\left(i\right)}{\mathrm{KT}}}=e^{-\frac{\Delta E}{KT}} & \left(others\right) \end{array}$$

In Equation (14), *E*(*i*) and *E*(*j*) represent the internal energy generated by the solid in two states, *i* and *j*. In turn, Δ*E*, *K*, and *T* represent the increment in internal energy, Boltzmann’s constant, and temperature of the material itself, respectively.

By analyzing the advantages and disadvantages of the two algorithms from a comprehensive perspective, a PSO algorithm based on simulated annealing is created, which enhances the particle swarm diversity, effectively suppresses the occurrence of premature convergence, and retains the advantages of the short local convergence time of the primary particle swarm algorithm, which can provide a sufficient guarantee for the accuracy of local search while improving the global search capability of the algorithm. The parameters of the SA algorithm were set as follows: the starting temperature was set to 1000 °C and the decay factor was 0.2. The parameters of the PSO algorithm were set as follows: the maximum number of runs was 100; the overall size of the species was 20; C1 and C2 were set to 2; and the inertia weight was set to 0.9. The SA algorithm can quickly determine the location of the solution in a short period and use it as its initial coordinates. It then uses the PSO algorithm for targeted screening to determine the optimal solution.

### 3.3. Construction of SAPSO-ELM Coal and Gas Outburst Prediction Model in Deep Coal Mines

When the ELM model is trained, its input layer weights and implied critical layer values are usually set unexpectedly and are not sufficiently stable. The built model is prone to several deficiencies, such as low prediction efficiency and weak generalization ability. Therefore, this study used the SAPSO algorithm to improve the ELM input layer weights and implied critical layer values to construct a SAPSO-ELM prediction model for coal–gas outburst prediction in deep coal mines. The steps of the optimization search were as follows.

Step 1: Randomly assign the training and test sets and normalize the data.

Step 2: Construct the network structure of the extreme learning machine, and then set the population size and number of particles.

Step 3: Set the parameters of the SAPSO algorithm.

Step 4: Record the optimal value of the current iteration.

Step 5: Determine when the error reaches the allowable range and when the number of runs reaches the idealized state. Stop the run when the ideal error disappears and start Step (7), and vice versa for Step (6).

Step 6: Update the particle velocity and position for iteration.

Step 7: Determine the optimal weights and thresholds.

Step 8: Train the whole test set and start the prediction.

## 4. Prediction Model Performance and Analysis of Experimental Results

### 4.1. KPCA-Based Feature Index Extraction

#### 4.1.1. Data Pre-Processing

In all the predictive indexes of coal–gas outbursts in deep coal mines, discretization between the magnitudes and orders of importance is produced. If the initial data are directly used, it will severely impact the stability and continuity of the model. Therefore, when studying the predictive indexes by KPCA, the normalized disposition operation of the initial data should be completed first, and the maximum–minimum method is selected for processing, with the specific formula as follows:

In the case of positively correlated indicators, also called benefit-based indicators, the following treatment is performed:

(15) $$x_{i}^{\prime }=\frac{x_{i}-x_{\min}}{x_{\max}-x_{\min}}$$

In the case of negatively correlated indicators, also referred to as cost-based indicators, the following treatment is performed:

(16) $$x_{i}^{\prime }=\frac{x_{\max}-x_{i}}{x_{\max}-x_{\min}}$$

In the above equations, *x*_max_ and *x*_min_ represent, in turn, the two extreme values of a certain influence factor in the data information series embedded within the prominent predictor system, *x*’*_i_* represents the predictor obtained by normalization, and *x_i_* represents the original indicator.

#### 4.1.2. Feature Extraction

After normalizing the original data, we selected the Gaussian radial basis kernel function with good nonlinear exploration ability for correlation analysis, where σ represents the hyperparameter of the kernel function, and was set to 500 based on relevant experience [38], and the eigenvalues of all the predictors with their contribution rates can be obtained through certain processing; the specific data are shown in Table 2. It is shown that the cumulative contribution rate of the first five indicators is 91.17%, according to the principal component extraction criterion, when the cumulative contribution rate of the extracted main components reaches 90% or more, they can represent all the information contained in the principal components, which can effectively solve the degradation problem of prediction accuracy caused by high-dimensional data input, and significantly improve the computational speed and prediction accuracy of the prediction model. Therefore, the first five indicators were used in this study to replace the initial 12 predictors, that is, the input variables were set as Y_1_, Y_2_, Y_3_, Y_4_, and Y_5_ in the model. The relevant data for some new variables after the feature extraction operation are listed in Table 3.

### 4.2. Training SAPSO-ELM Model Network Performance

The 178 initial datasets were randomly divided into 50 test sets and 128 training sets. ELM can achieve the learning purpose by adding only the number of hidden layers; the correspondence between the number of SAPSO-ELM hidden layer nodes and the test accuracy of the training set is shown in Table 4. For 8–35 hidden layer nodes, when the number of hidden layers reached 34, the test accuracy of the corresponding training set reached the highest value of 95.31%. Based on relevant experience, when the sigmoidal function was selected for the activation function, its generalization performance was the best, and the training error was the smallest [47]. Therefore, the author determined that the number of nodes in the implicit layer of the extreme learning machine was 34, and the network structure of the revolutionary learning machine was 5-34-4.

### 4.3. Comparison and Analysis of Prediction Results of Different Algorithms

**Figure 3:**
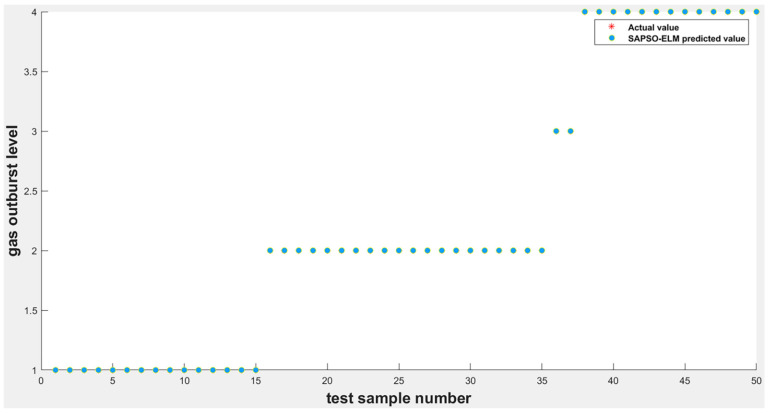
SAPSO-ELM model simulation output results.

After training the improved ELM model using the training samples, the degree of coal–gas outburst prediction of the test samples can be divided into four levels, based on the degree of damage produced by the coal–gas outburst phenomenon, which are indicated by 1, 2, 3, and 4. Thus, the overall distribution of the output image of the expected value is mainly point-like. The final output results may differ slightly if the system completes the prediction operation using multiple algorithm models, even for the same verification samples. Then, the comparison is performed between the difference and real data of the test samples to clearly evaluate the advantages and disadvantages of the algorithm. This test set was compared with the ELM and PSO-ELM algorithms to compare the prediction effect of each. The simulation results are shown in Figure 3; the accuracy of the SAPSO-ELM prediction model improved to 100%.

#### 4.3.1. Comparison of SAPSO-ELM and ELM Prediction Results

**Figure 4:**
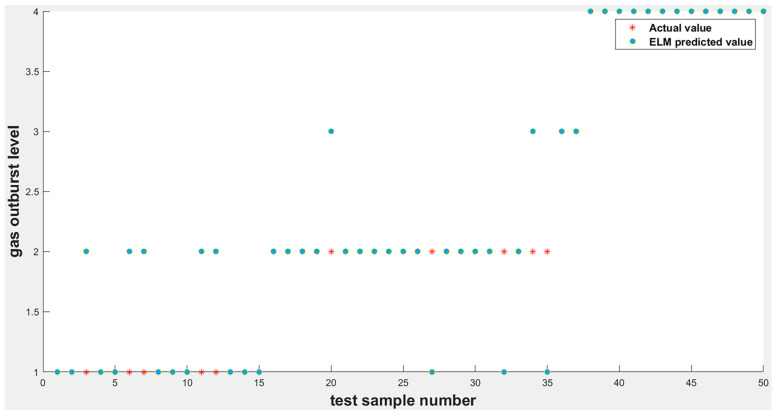
ELM model simulation output results.

To fully reflect the rationality of the ELM based on the improvement of the intelligent algorithm, this study was mainly simulated using MATLAB software, which is a commercial mathematics software produced by MathWorks of the United States. The ELM optimized without the intelligent algorithm was compared and analyzed with the ELM optimized using the SAPSO algorithm to verify its optimization effect. As shown in Figure 3 and Figure 4, when tested on 50 random samples, the optimized extreme-learning machine model showed no misclassification, and the accuracy rate was 100%. The model without optimization showed 10 misclassifications, with an accuracy rate of only 80%. Therefore, the prediction accuracy of the former was significantly higher than that of the other two. The biggest difference between these two algorithms is whether the SAPSO algorithm is used for the optimization. Compared with the unoptimized model, the method effectively overcomes the drawback of randomly selecting input weights and implicit threshold parameters in ELM. The accuracy of the model was significantly improved after the optimization operation, and it exhibited good generalization performance. Therefore, the improved ELM classification model was superior to the traditional ELM classification model.

#### 4.3.2. Comparison of SAPSO-ELM and PSO-ELM Prediction Results

**Figure 5:**
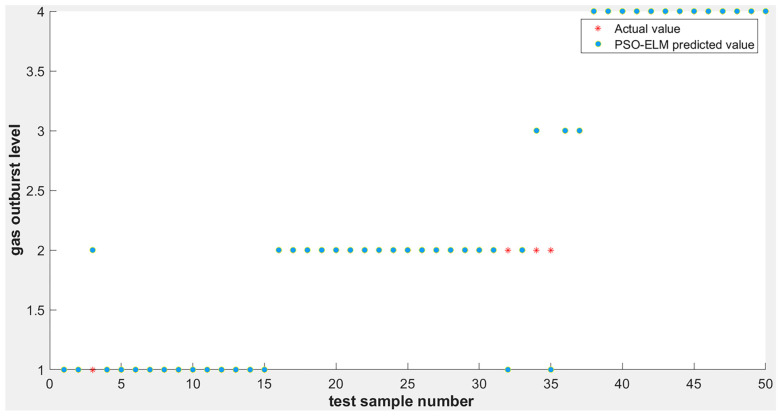
PSO-ELM model simulation output results.

As shown in Figure 3 and Figure 5, during the test, the SAPSO-ELM model did not produce misclassification and demonstrated an accuracy of 100%, whereas the PSO-ELM model produced four misclassifications with an accuracy of 92%. When predicting the 3rd, 32nd, 34th, and 35th datasets, the PSO-ELM predicts incorrect results and deviates from the actual results, while the SAPSO-ELM model can still predict the results correctly. This is because the particle swarm algorithm is more prone to the phenomenon of local optimum, which in turn generates premature convergence, and the simulated annealing particle swarm algorithm makes the particles deviate from the local optimum. Therefore, compared to the PSO-ELM model, the SAPSO-ELM model is more suitable for the required prediction.

#### 4.3.3. Comparison of Comprehensive Forecast Results

First, in terms of the time cost for model fitting, the ELM network training model adopts a forward single hidden layer structure; therefore, the joint weights w for the interaction between the input layer and the hidden layer, and the adjustment bias b for the hidden layer are obtained by initialization. This process is stochastic and requires no adjustment during the training process, however, only the number of neurons in the hidden layer should be set, and the output of the learning network can be determined by a one-step calculation; hence, the standard model is trained in a randomized manner. In this study, the standard ELM takes the shortest time (0.2175 s) superimposed on the PSO algorithm rules in the model training; this is because the particle swarm algorithm requires fewer adjustment parameter selection with more mature theoretical support and it is easy to implement. The training time of PSO-ELM is 3.3555 s, which is higher than the standard ELM. In the embedded SA computation mode, it is necessary to continuously perform optimization, judgment, and replacement among the training data. Therefore, the SAPSO-ELM algorithm model takes the most time for this prediction, reaching 4.3872 s.

Second, in the SAPSO-ELM algorithm, the ELM weights and thresholds are randomly generated, which may affect the accuracy of coal–gas outburst evaluation in deep coal mines. The PSO algorithm easily falls into local optimal solutions and produces premature convergence, and the most suitable ELM weights and thresholds are found by the SAPSO algorithm, such that the model generalization ability is enhanced to some extent.

As seen in Table 5, the SAPSO-ELM algorithm has a stronger generalization ability in both training and prediction accuracies than the unoptimized ELM and PSO-ELM prediction models. In terms of prediction time, the prediction speed of all three models was improved to different degrees after KPCA was adopted to extract the feature metrics. Although the SAPSO-ELM model requires a slightly longer time in the prediction phase than the other models, the accuracy and stability are more important in highlighting the prediction effect if the prediction time is within the specified interval. Overall, the advantages of the prediction model based on KPCA and SAPSO-ELM proposed in this study are more evident, mainly in the small training error, strong generalization ability, and good prediction accuracy, which can play a vital role in preventing and controlling coal–gas outburst disasters in deep coal mines when applied.

### 4.4. Testing the Effect of SAPSO-ELM Prediction Model

To verify the generalization performance of the SAPSO-ELM prediction model, the ROC curve, also known as the receiver operating characteristic curve [48], was used to evaluate the classifier validity, with values closer to the upper left corner of the curve indicating a better classification effect. When it is difficult to judge, it can be measured by the quantitative area under the curve (AUC) metric, with larger values indicating better classification effectiveness, in the range of 0.5 and 1 [49]. Gas outbursts are classified into four categories based on their outburst size, which is essentially a data classification problem, and a confusion matrix is introduced to evaluate the dichotomous classification problem in Table 6.

True/False represents whether the classification is correct, and Positive/Negative represents the category label of the predicted sample. Here, five indicators, *SEN*, *SPE*, *G*-mean, *F*-measure, and AUC, are selected to test the validity of the SAPSO-ELM prediction model, and the solution formula is as follows:

(17) SEN=TP/(TP+FN)

(18) SPE=TN/(TN+FP)

(19) PRE=TP/(TP+FP)

(20) $$G-\mathrm{mean}=\sqrt{SEN\times SPE}$$

(21) F−measure=2×SEN×PRE/(SEN+PRE)

**Figure 6:**
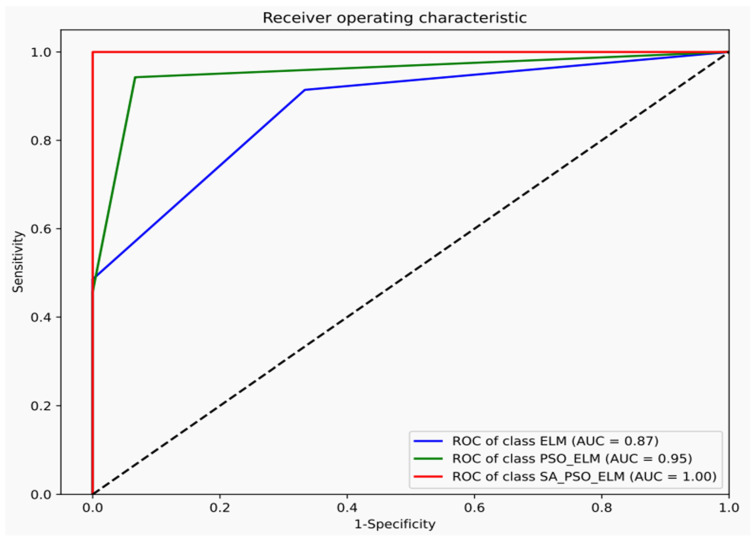
ROC curve of three groups of prediction models.

As shown in Figure 6, the classification curve of ELM is closest to the diagonal, its corresponding AUC is 0.87, and the corresponding AUC of PSO-ELM is 0.95. The SAPSO-ELM model proposed in this study corresponds to the largest AUC of up to one, which is in the ideal classification state. As presented in Table 7, the five metrics obtained by the calculation show that SAPSO-ELM is generally higher than the other two prediction models. Therefore, the improved ELM with the SAPSO is effective in improving the classifier performance.

## 5. Discussions

With the depletion of shallow coal resources, the mining intensity and depth of coal mines are increasing gradually. Moreover, the geological conditions of deep coal seams are becoming incredibly complex; the coal seam inclination changes significantly with many folds and faults. Under the combined effect of soft coal, low coal permeability, high stress, high gas content, and high gas pressure, coal seams show more complicated characteristics that lead to an increase in frequency and intensity of coal mine disasters. This also induces accidents, such as rockbursts, and coal and gas outbursts, posing a severe threat to mine safety. The aforementioned factors have led to new characteristics of gas outbursts in deep coal mines. In addition, the coupling between multiple hazards has led to a more complex mechanism of gas outbursts and requires an in-depth analysis of the conditions. The mechanism of gas outburst is an important prerequisite for effective prevention of this hazard risk. To further improve the accuracy of coal–gas outburst prediction in deep coal mines, this study improved the original gas outburst prediction index system based on an in-depth analysis of coal–gas outburst mechanisms and influencing factors, combined with examples of gas outburst accidents in deep coal mines and the existing literature on outburst prediction indices. A coal–gas outburst prediction model for deep coal mines was constructed based on KPCA and SAPSO-ELM algorithms. The prediction model was also applied to predict coal–gas outbursts from mine excavation studies, which provided a significant reference value for improving the safety of deep mine production.

Most of the existing literature contains information on shallow coal mining. Studies have been conducted on some of the influencing factors, however, these studies mostly ignore the complicated nonlinearity and correlation between these factors, which significantly affect the reliability of predicting gas outbursts in deep coal mines. With the continuous extension of coal mining depth, it is necessary to construct green, efficient, safe, and intelligent mines, and promote the process of neutralizing the carbon peaks; thus, it is of practical and theoretical significance to predict coal–gas outbursts in deep coal mines. In this study, we analyzed the coal–gas outburst hypothesis, considered the data accessibility to systematically understand the outburst-causing factors, and used the improved intelligent algorithm to make a scientific prediction. The example proved that the proposed model exhibits a good generalization performance, and can effectively prevent and address gas outburst disasters, which can guide coal mining enterprises to perform safe and effective deep coal mining.

Furthermore, with the increasing depth and intensity of coal seam mining, the geological conditions become more complex, coupling complexity between multiple disasters becomes higher, and corresponding influencing factors increase significantly. Therefore, in future research, it is necessary to widen the research on the process of coal–gas outbursts and related mechanisms in deep coal mining. Moreover, determining the coupling relationship between multiple coal-mining disasters and increasing the application of intelligent equipment in monitoring deep coal mine power disasters through numerous channels will further aid in continuous improvement in the management of coal mine enterprises, effective prevention and control of coal–gas outburst disasters, and realize safe and efficient mining in deep coal mines.

## 6. Conclusions

(1) In this study, we improved the original gas outburst prediction system by combining the examples of gas-outburst accidents in deep coal mines and the existing literature on gas outburst prediction indices, and constructed 12 deep mine gas outburst prediction indices, including coal seam mining depth, coal thickness variation coefficient, coal body damage type, coal seam lithology, mine temperature, soft partition thickness, gas pressure, coal seam gas content, coal seam resolvable gas content, coal seam permeability coefficient, coal body solidity coefficient, and gas discharge initial velocity. The 12 deep mine gas outburst prediction indices were analyzed by KPCA, and then reduced in dimensionality to solve the problem of input variables of the complex machine learning model and significantly improve the computational rate of the prediction model.

(2) We used the PSO algorithm to improve the ELM. Simultaneously, given the shortcomings of the PSO algorithm in terms of slow convergence speed and premature convergence, a prediction model of deep coal mine gas outburst based on SAPSO of the ELM was created. It fundamentally realizes the optimization of the prediction model of the ELM, enhances the generalization performance and stability of the model, and significantly improves the accuracy of gas outburst prediction in deep mines.

(3) The prediction accuracies of the SAPSO-ELM, PSO-ELM, and ELM models were 100%, 92%, and 80%, respectively, with training times of 4.3872 s, 3.3555 s, and 0.2175 s, respectively. We used MATLAB software to conduct relevant simulation tests and comparative studies on actual data of gas outbursts in deep coal mines. It was shown that the SAPSO-ELM model has the highest validity in determining the optimal global solution and can predict the gas outburst intensity of deep mines more accurately. In addition, although the SAPSO-ELM model takes slightly more time than the other models in the prediction process, its accuracy and stability are more critical in the outburst prediction effect if the prediction time is within a specified interval. Therefore, the gas outburst prediction model for deep coal mines proposed in this study has better generalization performance, and it analyzes and predicts deep coal mine gas outbursts from a new perspective, which is beneficial for ensuring safety in deep mines.

(4) The ROC curve was used to further test the proposed coal–gas outburst prediction model for deep coal mines. Its evaluation indices, such as SEN, SPE, G-mean, AUC, and F-measure, were significantly higher than those of other prediction models, which proved that the proposed prediction model has a good generalization ability. Its advantages are mainly reflected in its higher prediction accuracy and faster operation speed, which can be used for disaster prevention and control in deep coal mines.

## Figures and Tables

**Table 1:** Sample raw data of factors influencing coal–gas outburst in deep coal mines.

Number	X_1_	X_2_	X_3_	X_4_…X_9_	X_10_	X_11_	X_12_	Level
1	25	2.5	1	…	0.0063	6	1	2
2	32	2.5	1	…	0.0152	6.2	0.8	1
3	32	2.5	1	…	0.0013	9.5	0.7	1
4	32	2.5	1	…	0.0013	9.5	0.7	1
5	36	2	3	…	0.5596	6	1	1
6	32	2.5	1	…	0.0152	3.3	0.8	1
7	32	2.5	1	…	0.0013	5	0.7	1
8	25	2.5	1	…	0.0063	5.8	0.8	2
…	…	…	…	…	…	…	…	…
171	28.54	4.5	4	…	0.762	4.7	1.3	4
172	34.88	4.5	4	…	0.003	6.8	0.8	4
173	28.54	4.5	4	…	0.762	10	0.6	4
174	34.88	4.5	4	…	0.003	6.1	0.7	4
175	34.88	4.5	4	…	0.003	6.1	0.5	4
176	28.54	4.5	4	…	0.762	6.1	0.6	4
177	28.54	4.5	4	…	0.762	7.4	0.9	4
178	34.88	4.5	4	…	0.003	5	0.96	4

**Table 2:** Contribution rate of each principal component.

Number	Eigenvalue	Variance Contribution Rate	Cumulative Contribution Rate
1	9.5424	60.72	60.72
2	2.0395	12.98	73.7
3	1.3674	8.70	82.4
4	0.8078	5.14	87.54
5	0.5543	3.63	91.17
6	0.4145	2.74	93.91
7	0.2623	1.77	95.68
8	0.1949	1.34	97.02
9	0.1911	1.32	98.34
10	0.1218	0.87	99.21
11	0.0580	0.47	99.68
12	0.0349	0.32	100

**Table 3:** Sample data after feature extraction.

Number	Y1	Y2	Y3	Y4	Y5
1	−0.188	−0.008	−0.087	0.002	−0.014
2	−0.151	0.025	−0.063	0.007	0.037
3	−0.098	0.094	−0.044	−0.017	0.139
4	−0.098	0.095	−0.043	−0.019	0.138
5	0.008	−0.019	−0.013	−0.295	−0.032
6	−0.122	0.017	−0.046	0.012	−0.013
7	−0.110	0.078	−0.038	−0.022	0.024
8	−0.173	0.038	−0.078	−0.032	−0.011
9	−0.122	0.128	−0.061	−0.045	0.019
10	−0.101	0.071	0.084	−0.106	0.023
11	−0.109	0.054	0.081	−0.100	0.049
12	−0.121	0.128	−0.060	−0.044	0.018
13	−0.123	0.129	−0.066	−0.048	0.084
14	−0.152	0.014	−0.061	0.012	0.017
15	−0.153	0.047	−0.056	−0.011	0.014
16	−0.096	0.077	0.086	−0.106	0.034
17	−0.150	0.012	−0.059	0.014	0.016
18	−0.114	0.068	−0.039	−0.017	0.046
19	−0.151	0.035	−0.054	−0.004	0.008
20	−0.163	0.017	−0.051	−0.004	−0.071

**Table 4:** Relationship between node number and accuracy of SAPSO-ELM training set.

Node Numbers	Accuracy
8	0.7813
9	0.8125
10	0.7734
11	0.8672
12	0.8203
13	0.8984
14	0.8359
15	0.8281
16	0.9063
17	0.8359
18	0.8750
19	0.8906
20	0.8984
21	0.9219
22	0.9297
23	0.9297
24	0.9297
25	0.9219
26	0.9219
27	0.9375
28	0.9375
29	0.9297
30	0.9297
31	0.9375
32	0.9297
33	0.9375
34	0.9531
35	0.9297

**Table 5:** Prediction effect of each model after KPCA dimensionality reduction.

Indicators	PSO-ELM	ELM	SAPSO-ELM
Prediction accuracy	92	80	100
Training accuracy	96	92	100
Time/s	3.3555	0.2175	4.3872

**Table 6:** Confusion matrix.

Prediction		
Practice	True Positive (*TP*)	True Negative (*TN*)
	False Positive (*FP*)	False Negative (*FN*)

**Table 7:** Performance indicators of three groups of prediction models.

Algorithms	SEN	SPE	AUC	G-Mean	F-Measure
ELM	0.88	0.67	0.87	0.59	0.77
PSO-ELM	0.92	0.93	0.95	0.86	0.93
SAPSO-ELM	1	1	1	1	1

## Data Availability

Not applicable.
